# Goitre ovarien bénin: à propos d'un cas et revue de la littérature

**DOI:** 10.11604/pamj.2015.21.271.7209

**Published:** 2015-08-11

**Authors:** Boutaina Lachiri, Zineb Benkerroum, Mohamed Allaoui, Zazi Abdelgheni, Khalid Guelzim, Jaouad Kouach, Driss Moussaoui Rahali, Mohamed Dehayni

**Affiliations:** 1 Service de Gynécologie-Obstétrique, Hôpital Militaire d'Instruction Med V, Maroc; 2 Service d'Anatomie Pathologique, Hôpital Militaire d'Instruction Med V, Maroc

**Keywords:** Struma ovarii, goitre ovarien, thyroïde, tératome ovarien, histopathologie, Struma ovarii, ovarian goiter, thyroid, ovarian teratoma, histopathology

## Abstract

Goitre ovarien appartient au groupe des tératomes mono dermiques. C'est une variété rare des tumeurs de l'ovaire. L'examen histopathologique est indispensable pour le diagnostic de cette entité. Le goitre ovarien peut être à l'origine d'anomalies du bilan thyroïdien. Vu le risque de la transformation maligne le traitement chirurgical reste la seule alternative thérapeutique. Notre patiente avait une grande masse annexielle unilatérale, multi-lobulée faisant 14,45 x 12,21 cm sans signes d'hyperthyroïdies associées. Un traitement conservateur par kystectomie a été réalisé. L'examen anatomopathologique a confirmé le diagnostic d'un goitre ovarien. Les suites poste opératoires étaient simples et le suivi clinique n'a pas montré d'anomalies avec un recul de 9 mois.

## Introduction

Le goitre ovarien ou Struma ovarii est caractérisé par la présence de plus de 50% du tissu thyroïdien dans une tumeur ovarienne, généralement considérée comme tératome mono dermique mature. C'est une tumeur relativement rare qui représente 1% de toutes les tumeurs ovariennes et 3% de toutes les tumeurs dermoïdes de l'ovaire [1]. La plupart des cas de Struma ovarii sont bénins et généralement unilatéraux, seulement 5% à 10% de Struma ovarii sont malins (goitre ovarien malin), accompagnés exceptionnellement de métastases. Malgré que le tissu thyroïdien est majoritaire dans ces tumeurs, l′hyperthyroïdie est vu dans environ 8% des patientes atteintes de goitre ovarien [1]. Nous rapportons un cas de goitre ovarien bénin chez une patiente de 29 ans. À travers notre observation, une revue de la littérature a été faite pour mieux cerner les aspects épidémiologiques, diagnostiques, histologiques et thérapeutiques de cette entité clinique rare.

## Patient et observation

**Figure 1:**
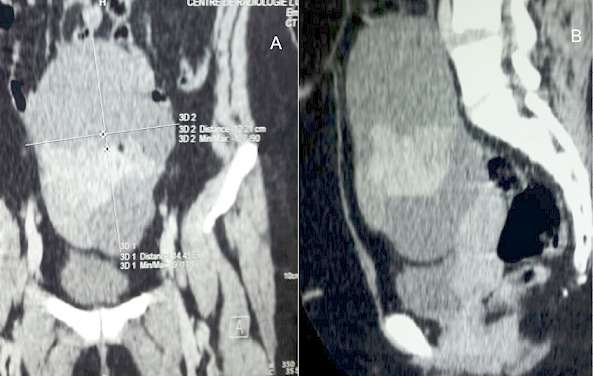
Tomodensitométrie pelvienne montrant une masse sus utérine à double composante charnue et kystique de 14,45 /12,21 cm de diamètre en faveur d'un kyste ovarien

**Figure 2:**
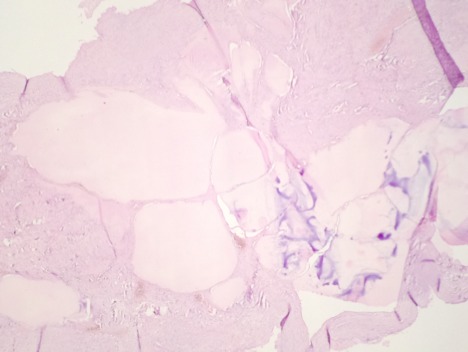
Lésion tératomateuse mono dermique faite d'un tissu thyroïdien (HE, x100)

**Figure 3:**
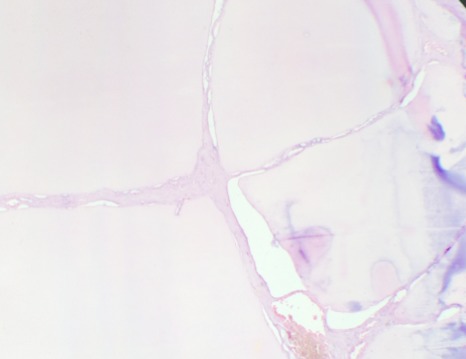
Il est fait de vésicules thyroïdiennes souvent kystisées, tapissées de cellules folliculaires aplaties (HE, x400)

Mme A.H âgée de 29 ans, deuxième geste deuxième pare avec deux enfants vivants, ses accouchements étaient par voie basse. Sans antécédents personnels ou familiaux pathologiques notables. Son cycle menstruel est régulier sous contraception orale au oestroprogestatifs depuis 6 ans. Elle a consulté au service de gynécologieobstétrique pour des douleurs pelviennes modérées intermittentes évoluant depuis deux mois sans notion de saignement, leucorrhées ni signes urinaires ou digestifs associés. L'examen général trouve une patiente en bon état général, l’état hémodynamique stable avec un IMC à 24 kg/m^2^. L'examen gynécologique trouve un col d'aspect sain le touché vaginal combiné au palper abdominal trouve une masse abdomino-pelvienne arrivant à 2 travers de doigts au dessous de l'ombilic, bien limitée de consistance molle, mobile et légèrement sensible. Le reste de l'examen clinique est sans particularité. Par ailleurs, la patiente ne présentait pas des signes d'hyperthyroïdie. La patiente a bénéficié d'une échographie pelvienne qui a montré une image kystique hétérogène multi-loculée à septas fines et épaisses faisant 12/10 cm. Une tomodensitométrie pelvienne a montré une masse sus utérine à double composante charnue et kystique de 14,45 /12,21 cm en faveur d'un kyste ovarien (Figure 1). Les marqueurs tumoraux sont revenus normaux (CA125, ACE, CA19-9 et BHCG). La patiente a bénéficié d'une laparotomie. L'exploration préopératoire montre une volumineuse masse ovarienne kystique droite polylobée, multi-cloisonnée, à paroi fine régulière et hyper-vascularisée sans épanchement péritonéal. Le reste de l'exploration abdominale est sans particularité. Les opérateurs ont procédé à une kystectomie idéale avec préservation du parenchyme ovarien droit. Les suites postopératoires étaient simples. L'aspect macroscopique à l'examen anatomopathologique trouve à la coupe, une cavité multiloculaire à paroi fine remplie d'un liquide séreux et d'un matériel gélatineux. Sur le plan microscopique, il s'agit d'un tératome mono dermique, fait essentiellement de tissu thyroïdien en faveur d'un goitre ovarien sans critères de malignité (Figure 2, Figure 3). Un bilan thyroïdien demandé à postériori est revenu normal. L'examen clinique à 9 mois est sans particularité.

## Discussion

Les tératomes sont des tumeurs de la lignée germinale composées de cellules dérivées d'une ou plusieurs des trois couches embryonnaires (méso-, endo- et ectoderme), qui peuvent être uniou pluritissulaires [2]. Décrits pour la première fois par Von Kalden en 1895, les goitres ovariens (ou struma ovarii) sont des tératomes uni-tissulaires de l'ovaire composés majoritairement (plus de 50% de la tumeur) voire exclusivement (goitre ovarien pur) de tissu thyroïdien [3]. L′âge de survenue de Struma ovarii est compris entre 30 et 50 ans, mais des cas ont été rapportés chez des femmes post-ménopausées et exceptionnellement cette tumeur peut se produire chez des filles pré pubères [1].

Le plus souvent, la symptomatologie est pauvre, le diagnostic de masse ovarienne étant fait soit par un examen systématique, soit devant l'existence de douleurs pelviennes, d'une masse abdominale ou de troubles du cycle [4]. Environ 5 à 8% des goitres ovariens s'accompagneraient d'hyperthyroïdie [3]. En effet, comme dans la glande cervicale, le tissu thyroïdien ectopique peut également s'autonomiser [5]. En plus des signes et symptômes habituels d′une masse pelvienne, l'ascite survient dans un tiers des cas, et parfois certaines patientes peuvent montrer des caractéristiques du syndrome de Meigs [1].

Le diagnostic préopératoire est très difficile en raison de différents types de tératomes avec des caractéristiques similaires. Les caractéristiques échographiques de goitre ovarien sont également non spécifiques, on peut retrouver une grosse tumeur hétérogène mixte solide et liquide, avec cloisons et végétations [2]. Néanmoins, le flux doppler peut aider au diagnostic préopératoire de goitre ovarien. Les signaux de débit sanguin, détectés à partir du centre de la lésion échogène, et une faible résistance à l′écoulement peuvent être plus fréquents dans le goitre ovarien en raison du caractère richement vascularisé du tissu thyroïdien comparativement aux autres composants des tératomes non vascularisés tels que la graisse ou les phanères [6]. L′utilisation des caractéristiques des tératomes de l′ovaire à l′échographie, TDM et l′IRM peut aider dans la différenciation et le diagnostic. Kim et al ont évalué les caractéristiques radiologiques des goitres ovariens en IRM, il s'agit d′une masse complexe multi-lobulée avec cloisons épaissies, kystes multiples d'intensités de signal variables avec des composants solides [7]. Les portions solides, qui se rehaussent fortement après injection de gadolinium, correspondent au tissu thyroïdien et au stroma contenant d'abondants vaisseaux sanguins et du tissu fibreux. En dehors de métastases, il n'existe pas de critères radiologiques de malignité du goitre ovarien. Seulement une scintigraphie pelvienne préopératoire à l′iode radioactif (131I) pourrait montrer un tissu thyroïdien actif [7]. L'aspect macroscopique caractéristique des goitres ovariens est celui d'une tumeur à composante mixte, solide et kystique (à contenu muqueux ou gélatineux), de couleur marron vert, généralement de grande taille et associée aux autres composants d'un tératome mature dans près de la moitié des cas [8]. L'aspect microscopique retrouve des inclusions de follicules thyroïdiens contenant du colloïde qui sont soit encapsulées, soit irrégulièrement distribuées parmi les autres composants du tératome [8]. L'architecture tissulaire est tout aussi variée que celle de la glande thyroïde, avec des structures macro ou micro-vésiculaires, de goitre colloïde, pseudo tubaires ou trabéculaires, qui peuvent être associées entre elles. Les cellules peuvent présenter un cytoplasme éosinophile ou clair, parfois vacuolé [2]. Le diagnostic histologique des formes malignes du goitre ovarien a longtemps été controversé et mal évalué en raison de l'absence de critères diagnostiques uniformes et de la rareté de la tumeur [9].

En cas de goitre ovarien bénin, aucun traitement complémentaire à l'ovariectomie unilatérale ou à une exérèse simple n'est nécessaire [5]. Une hyperthyroïdie éventuelle initiale peut exister dans 15% des struma ovarii, elle cesse après l'exérèse tumorale. Parfois, un goitre ovarien sécrétant peut mettre au repos l'axe hypothalamohypophysaire avec risque d'hypothyroïdie postopératoire. Quelques cas de goitre ovarien avec manifestations d'auto-immunité de type maladie d'Hashimoto ou maladie de Basedow ont été rapportés [5].

## Conclusion

La présence majoritaire de tissu thyroïdien au sein d'un tératome de l'ovaire est appelée traditionnellement goitre ovarien et n'en représente qu'une forme clinique particulière, le plus souvent bénigne et d'excellent pronostic, même si elle peut, de façon exceptionnelle, présenter une dégénérescence maligne dont le traitement et le suivi miment ceux du carcinome thyroïdien. Pour les patientes atteintes de goitre ovarien bénin, les normes chirurgicales suivies sont suffisantes et l'histopathologie est l'outil de confirmation du diagnostic.
